# Increased salivary microvesicles are associated with the prognosis of patients with oral squamous cell carcinoma

**DOI:** 10.1111/jcmm.14291

**Published:** 2019-03-25

**Authors:** Wen‐Qun Zhong, Jian‐Gang Ren, Xue‐Peng Xiong, Qi‐Wen Man, Wei Zhang, Lu Gao, Chen Li, Bing Liu, Zhi‐Jun Sun, Jun Jia, Wen‐Feng Zhang, Yi‐Fang Zhao, Gang Chen

**Affiliations:** ^1^ The State Key Laboratory Breeding Base of Basic Science of Stomatology (Hubei‐MOST) & Key Laboratory of Oral Biomedicine Ministry of Education, School & Hospital of Stomatology Wuhan University Wuhan China; ^2^ Department of Oral and Maxillofacial Surgery, School & Hospital of Stomatology Wuhan University Wuhan China

**Keywords:** apoptosis, biomarker, microvesicles, oral squamous cell carcinoma, saliva

## Abstract

Microvesicles (MVs), which are cell‐derived membrane vesicles present in body fluids, are closely associated with the development of malignant tumours. Saliva, one of the most versatile body fluids, is an important source of MVs. However, the association between salivary MVs (SMVs) and oral squamous cell carcinoma (OSCC), which is directly immersed in the salivary milieu, remains unclear. SMVs from 65 patients with OSCC, 21 patients with oral ulcer (OU), and 42 healthy donors were purified, quantified and analysed for their correlations with the clinicopathologic features and prognosis of OSCC patients. The results showed that the level of SMVs was significantly elevated in patients with OSCC compared to healthy donors and OU patients. Meanwhile, the level of SMVs showed close correlations with the lymph node status, and the clinical stage of OSCC patients. Additionally, the ratio of apoptotic to non‐apoptotic SMVs was significantly decreased in OSCC patients with higher pathological grade. Consistently, poorer overall survival was observed in patients with lower ratio of apoptotic to non‐apoptotic SMVs. In conclusion, the elevated level of SMVs is associated with clinicopathologic features and decreased survival in patients with OSCC, suggesting that SMVs are a potential biomarker and/or regulator of the malignant progression of OSCC.

## 1 INTRODUCTION

Oral squamous cell carcinoma (OSCC) is the most common subtype of head and neck squamous cell carcinoma.^1^ Although the treatment of OSCC has made progresses recently, the prognosis of OSCC is still poor, with high rate of local recurrence and a 5‐year survival rate at 50%.^2^ Thus, more mechanisms behind the development of OSCC need to be unmasked.

Microvesicles (MVs) are submicron (100‐1000 nm) membrane vesicles secreted by cells.^3^ In addition to various biological functions, MVs also possess significant potential to serve as biomarkers for malignant tumours.^4^ It has been well‐established that MVs are present and elevated in the blood of patients with various diseases, including diabetes, cardiovascular diseases and cancers.^5^, ^6^, ^7^ Our previous studies have shown that the level of circulating MVs (CMVs) in peripheral blood is significantly elevated in patients with OSCC compared to healthy donors, and it is positively correlated with the clinical features of OSCC patients.^8^, ^9^

In addition to the plasma, increased levels of MVs have been reported in other types of body fluids, such as urine in patients with bladder cancer,^10^ ascites in patients with ovarian carcinoma^11^ and pleural fluids in patients with lung cancer.^12^ Previous studies have confirmed that MVs are present in the saliva of healthy donors.^13^ However, the change in salivary MVs (SMVs) during the development of OSCC is still unclear. Since the primary tumour is directly immersed in the salivary milieu in OSCC patients, tumour cells may potentially release MVs into the saliva. Moreover, previous studies have demonstrated that MVs produced upon cell apoptosis (known as apoptotic MVs) or cell activation might possess different pathophysiological functions.^14^, ^15^, ^16^ Because suppression of apoptosis plays an important role in the development of cancer, the level of apoptotic MVs,^17^, ^18^ which is usually a reflection of the level of cell apoptosis, has been linked to the development of cancer.^19^, ^20^, ^21^ For instance, a previous study showed that the level of apoptotic MVs in peripheral blood possessed diagnostic significance in lung cancer.^22^ However, the biomarker potential of SMVs and apoptotic SMVs in OSCC patients remains to be clarified.

In the present study, the level of total SMVs and the proportion of apoptotic SMVs were evaluated and compared among healthy donors, patients with oral ulcer (OU) and patients with OSCC. In addition, the correlations of SMVs with the clinicopathologic features and prognosis of OSCC patients was examined.

## 2 MATERIALS AND METHODS

### 2.1 Isolation of SMVs and CMVs

This study was carried out according to the guidelines approved by the review board of the ethics committee of the Hospital of Stomatology, Wuhan University. Meanwhile, the study was performed according to the World Medical Association Declaration of Helsinki and the National Institutes of Health guidelines regarding to the use of clinical tissues. Written informed consent was obtained from all the subjects in our research. The volunteers were required to stop from performing oral hygiene procedures, smoking, drinking and eating at least 1 hour before saliva collection. Unstimulated saliva sample (5 mL) was collected between 9 AM and 10 AM according to a previously established protocol.^23^ For CMVs collection, 5 mL of venous citrated blood from OSCC patients or healthy donors was obtained. Then SMVs and CMVs from healthy donors, OU patients and OSCC patients were purified by a standard differential centrifugation protocol^8^, ^24^, ^25^ as described in the supplementary materials.

### 2.2 Characterization and quantification of SMVs and CMVs

According to our previous studies,^8^, ^24^ transmission electron microscopy (TEM), dynamic light scattering, carboxyfluorescein succinimidyl ester (CFSE) labeling and flow cytometry were first performed to characterize the purified SMVs and CMVs. Then, the concentrations of MVs were analysed by the count of “events” using flow cytometer. Briefly, identical volumes of a MV sample and the Flow‐count fluorospheres (Beckman Coulter; 10 μm in diameter) were mixed for detection. Since Flow‐count fluorospheres have a known concentration, the concentrations of MVs can be calculated as the following formula: (total number of events for the sample/total number of events for Flow‐count fluorospheres) × Flow‐Count fluorospheres assayed concentration. Following fluorochrome‐coupled antibodies and their corresponding isotypes were used for the analyses of MV phenotype: phycoerythrin (PE) labeled anti‐Annexin V, PE labeled anti‐CD31, FITC labeled anti‐CD41b, PE labeled anti‐EpCAM. All these antibodies were purchased from BD Biosciences. With regard to incubation buffers, Annexin V Binding Buffer containing calcium (BD Biosciences) was used for PE‐labeled Annexin V, and PBS was used for other antibodies recognizing cellular origin markers.

### 2.3 Immunohistochemistry

Based on previously described procedures,^26^, ^27^, ^28^ the samples from OSCC patients were fixed in 4% paraformaldehyde and embedded in paraffin. Immunohistochemistry was performed as described in the supplementary materials.

### 2.4 Statistical analysis

All data in our study were shown as Mean ± SD of three independent experiments. Student's *t* test, one‐way analysis of variance and Spearman's rank correlation test were used for statistical analysis. A value of *P* < 0.05 was considered statistically significant.

## 3 RESULTS

### 3.1 Clinical characteristics and follow‐up of the patients with OSCC

In our present study, the age distribution and gender ratio were similar among the healthy donors, patients with OU and patients with OSCC, with no significant difference (*P* > 0.05). All cases of OSCC patients were free of any other systemic disease including diabetes and cardiovascular disorders. Up to April 2018, the 65 OSCC patients were followed up from 2 to 45 months. The overall survival rate was 84.6%, the disease‐free survival rate was 76.9% and the recurrence rate was 21.5%.

### 3.2 The morphology, size and zeta potential of SMVs are in line with the features of MVs and are not changed in OSCC patients

**Figure 1:**
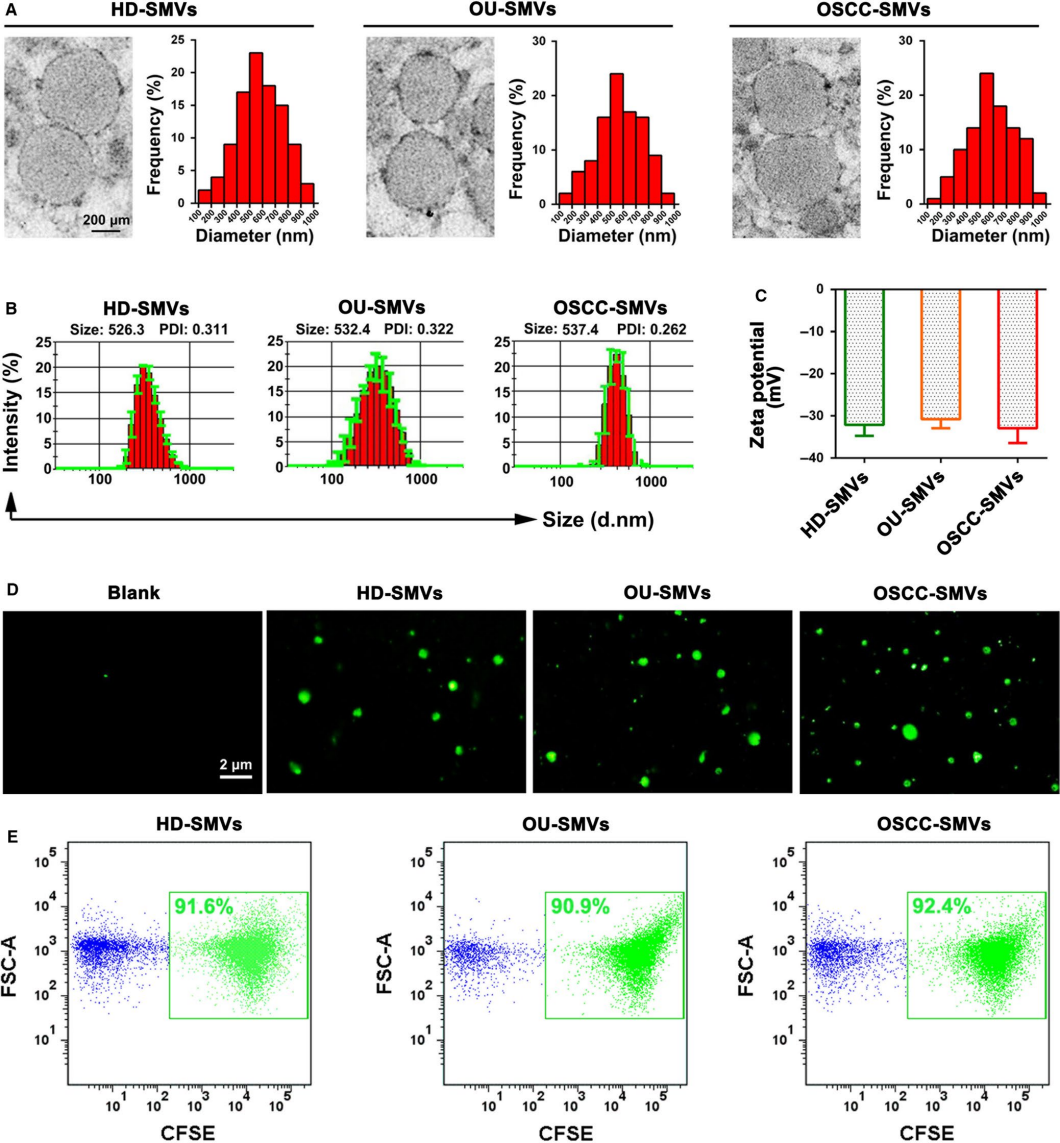
Characterization of salivary microvesicles (SMVs) derived from healthy donors (HD), patients with oral ulcer (OU) and patients with oral squamous cell carcinoma (OSCC). (A) Transmission electron microscopy (TEM) images of SMVs purified from healthy donors (HD‐SMV), OU patients (OU‐SMVs) and OSCC patients (OSCC‐SMVs). The size distribution of SMVs was analysed based on the TEM images. (B) Dynamic light scattering was performed to assess the size distribution of SMVs derived from healthy donors (HD‐SMV), OU patients (OU‐SMVs) and OSCC patients (OSCC‐SMVs). (C) Dynamic light scattering was carried out to detect the zeta potential of SMVs derived from healthy donors (HD‐SMV), OU patients (OU‐SMVs) and OSCC patients (OSCC‐SMVs). (D) Fluorescence images showing the labeling of SMVs by the fluorescent dye carboxyfluorescein succinimidyl ester (CFSE). (E) Representative flow cytometry dot‐plots showing the percentages of CFSE‐labeled SMVs. Data were expressed as Mean ± SD

As revealed by TEM images (Figure 1A), SMVs purified from healthy donors (HD‐SMV), OU patients (OU‐SMVs) and OSCC patients (OSCC‐SMVs) were membrane‐limited vesicles presenting round or slightly elongated shapes with diameters between 100 nm and 1000 nm. The flow cytometry (Figure S1) and dynamic light scattering (Figure 1B) analyses demonstrated that the purified SMVs had a size distribution ranging from 100 nm to 1000 nm, showing no significant difference among healthy donors, OU patients and OSCC patients. Moreover, the zeta potentials of HD‐SMV, OU‐SMVs and OSCC‐SMVs showed the similar distribution, ranging from −28 mV to −40 mV (Figure 1C). Additionally, the CFSE labeling assay showed that the purified SMVs could be successfully labeled by CFSE, indicating their membrane‐bound structures (Figure 1D). The flow cytometric results further showed that the majority of particles within the SMV samples were positive for CFSE staining (Figure 1E), suggesting the purity of the isolated SMVs. These above data demonstrate that the purified SMVs are in line with the features of MVs, and some of the basic properties of SMVs, such as physical morphology, size distribution and zeta potential, are not significantly changed in OSCC patients.

### 3.3 The level of SMVs is elevated in OSCC patients

**Figure 2:**
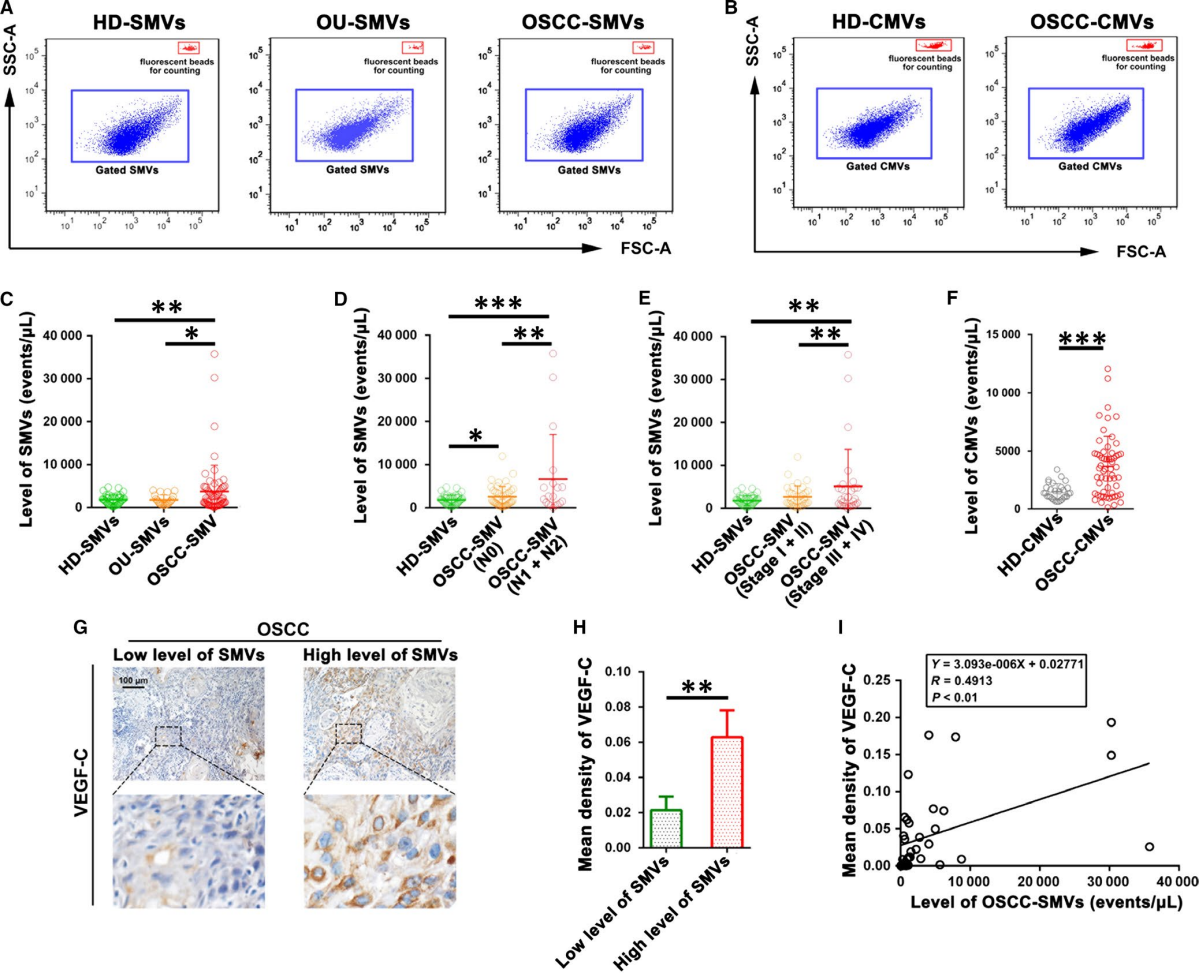
Quantification of the level of salivary microvesicles (SMVs) and circulating microvesicles (CMVs) in healthy donors (HD), patients with oral ulcer (OU) and patients with oral squamous cell carcinoma (OSCC). (A) Representative flow cytometry dot‐plots showing the gated SMVs (blue window) purified from healthy donors (HD‐SMV), OU patients (OU‐SMVs) and OSCC patients (OSCC‐SMVs) as well as the fluorescent beads for counting (red window). (B) Representative flow cytometry dot‐plots showing the gated CMVs (blue window) purified from healthy donors (HD‐CMVs) and OSCC patients (OSCC‐CMVs) as well as and the fluorescent beads for counting (red window). (C) Comparison of the levels of SMVs in healthy donors (HD‐SMV), patients with oral ulcer (OU‐SMVs) and patients with oral squamous cell carcinoma (OSCC‐SMVs). (D) Comparison of the levels of SMVs in healthy donors (HD‐SMV) and OSCC patients with or without lymphatic metastasis. (E) Comparison of the levels of SMVs in healthy donors (HD‐SMV), and OSCC patients with lower clinical stage (Stage I + II) or higher clinical stage (Stage III + IV). (F) Comparison of the levels of CMVs in healthy donors (HD‐CMVs) and patients with oral squamous cell carcinoma (OSCC‐CMVs). (G) Representative immunostaining of VEGF‐C in OSCC patients with different levels of SMVs. (H) Comparison of the levels of VEGF‐C in OSCC patients with different levels of SMVs. (I) Spearman's rank correlation test was performed to determine the correlation between the level of SMVs and the intratumoral expression level of VEGF‐C in OSCC patients. Data were shown as the Mean ± SD. *P < 0.05, **P < 0.01, ***P < 0.001

For quantifying the SMVs and CMVs, flow cytometry using flow‐count fluorospheres was performed. Representative images of flow cytometry for detecting SMVs and CMVs were shown in Figure 2A,B respectively. The fluorescent beads for counting were shown as the gated dot‐plots in the red window, while the SMVs or CMVs were shown in the blue window. The quantification results showed that the number of SMVs was ranged from 147 to 4704 events/μL (Mean ± SD: 1801 ± 1172 events/μL) in healthy donors, ranged from 523 to 4041 events/μL (Mean ± SD: 1791 ± 1207 events/μL) in patients with OU, and ranged from 65 to 35774 events/μL (Mean ± SD: 3758 ± 6119 events/μL) in patients with OSCC. Significant differences were found between OSCC patients and OU patients or healthy donors in terms of the level of SMVs (Figure 2C). In addition, the result also showed that the OSCC cases presenting as a non‐healing ulcer had higher levels of SMVs compared with the OU cases (Figure S2, *P* < 0.001). However, there was no significant difference between healthy donors and patients with OU in the level of SMVs (Figure 2C). Moreover, as shown in Figure 2D, OSCC patients with or without lymphatic metastasis showed an obviously higher level of SMVs than the healthy donors. Meanwhile, OSCC patients with higher clinical stage (Stage III + IV) had significantly higher level of SMVs than the healthy donors (Figure 2E, *P* < 0.01). Our previous studies have revealed that the level of CMVs in peripheral blood is also significantly increased in OSCC patients when compared with healthy donors. Here, we further investigated the potential correlation or difference in the levels of SMVs and CMVs in OSCC patients. As shown in Figure 2F, the level of CMVs was significantly elevated in OSCC patients compared to healthy donors (*P* < 0.001). Nevertheless, there was no significant correlation between the increased levels of SMVs and CMVs in OSCC patients (Figure S3A). In addition, our results revealed that the majority of OSCC‐CMVs were CD31^+^CD41b^+^ platelet‐derived MVs, while the majority of OSCC‐SMVs were positive for EpCAM, indicating the origin of epithelial cells (Figure S3B). These results demonstrate that the level of SMVs is elevated concomitant with the level of CMVs in patients with OSCC. However, the cellular origins of SMVs and CMVs in OSCC patients might be significantly different.

### 3.4 Increased SMVs are correlated with the clinicopathologic features of OSCC patients

Next, associations between the elevated level of SMVs and the clinicopathologic features of OSCC patients were investigated. As shown in Figure 2D, the level of SMVs in OSCC patients with lymph node metastasis was markedly up‐regulated compared to patients without lymph node metastasis (*P* < 0.01). Moreover, as shown in Figure 2E, the level of SMVs in patients with higher clinical stage (Stage III + IV) was obviously elevated when compared to that in patients with lower clinical stage (Stage I + II, *P *< 0.01). To verify the association between increased SMVs and the lymph node metastasis of OSCC, we then evaluated the correlation of the level of SMVs with the expression level of vascular endothelial growth factor C (VEGF‐C), one of the major pro‐lymphangiogenic growth factors, in the tumour samples of OSCC patients. The representative immunohistochemical images are shown in Figure 2G. The semi‐quantitative results revealed that the expression level of VEGF‐C in the tumour samples was markedly increased in the patients with higher levels of SMVs when compared to those with lower levels of SMVs (Figure 2H). Importantly, the correlation analysis showed that the intratumoral expression level of VEGF‐C in OSCC patients was positively associated with the level of SMVs (Figure 2I). These above results suggest that the increased SMVs could be a potential biomarker of clinicopathologic features of OSCC patients.

### 3.5 The ratio of apoptotic to non‐apoptotic SMVs is decreased in patients with poorly differentiated OSCC

**Figure 3:**
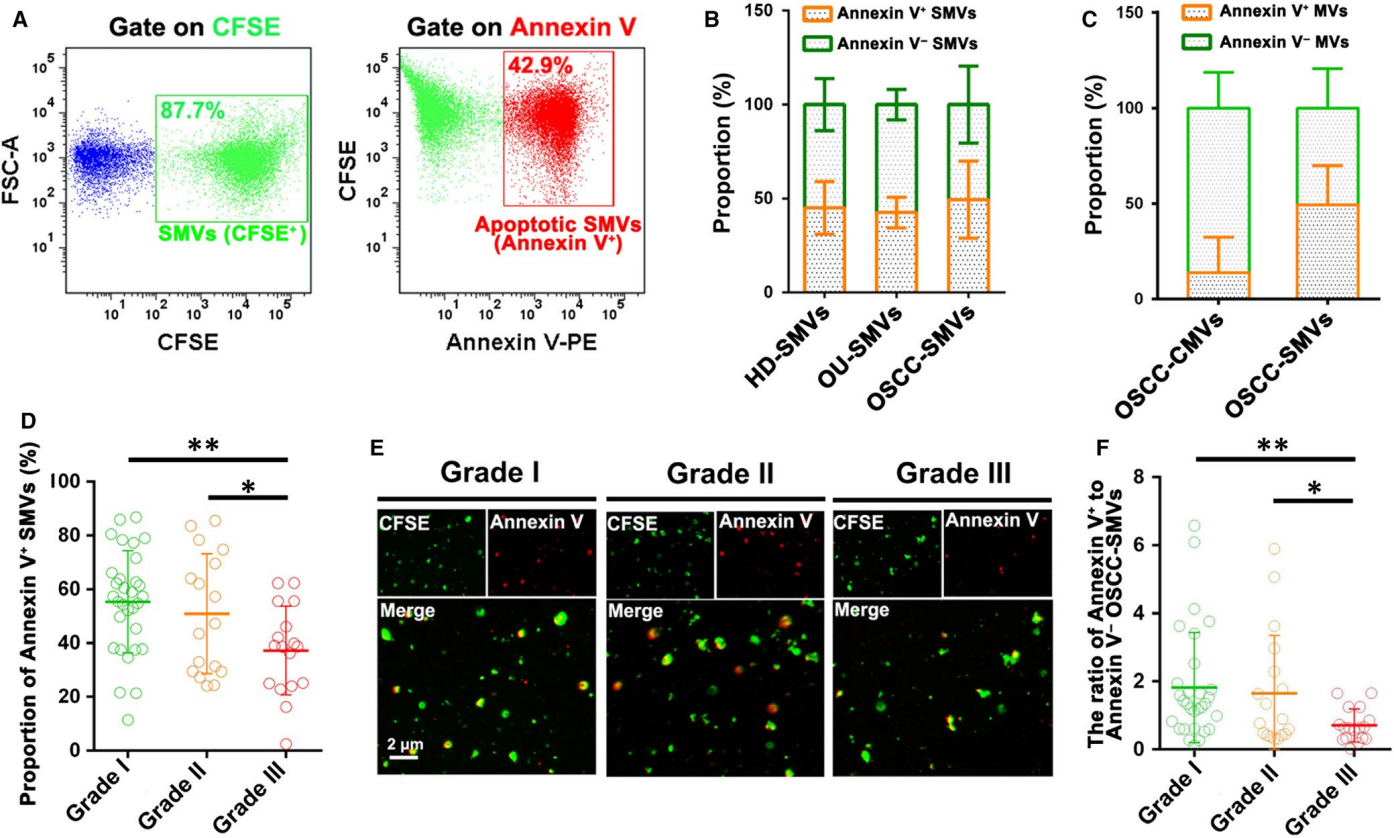
Quantitative analysis of the ratio of apoptotic to non‐apoptotic salivary microvesicles (SMVs). (A) Representative flow cytometry dot‐plots showing the gating strategy for apoptotic SMVs. (B) Quantitative analysis of the proportion of apoptotic and non‐apoptotic SMVs in healthy donors (HD‐SMV), patients with oral ulcer (OU‐SMVs) and patients with oral squamous cell carcinoma (OSCC‐SMVs). (C) Quantitative analysis of the proportion of apoptotic and non‐apoptotic OSCC‐CMVs and OSCC‐SMVs. (D) Quantitative analysis of the proportion of apoptotic SMVs in OSCC patients with different pathological grades. (E) Fluorescence images showing apoptotic SMVs (CFSE^+^Annexin V^+^) and non‐apoptotic SMVs (CFSE^+^Annexin V^−^) in OSCC patients with different pathological grades. (F) Quantitative analysis of the ratio of Annexin V^+^ to Annexin V^−^ OSCC‐SMVs in OSCC patients with different pathological grades. Data were expressed as Mean ± SD. *P < 0.05, **P < 0.01

Annexin V has been frequently used as a marker of MVs from apoptotic cells, especially when analysing the MVs in human body fluids.^6^, ^29^, ^30^ As shown in Figure 3A, the percentages of apoptotic and non‐apoptotic OSCC‐SMVs were analysed based on gating of CFSE (indicative of SMVs) and Annexin V (indicative of apoptotic origin). The results showed that the percentages of apoptotic (CFSE^+^Annexin V^+^) SMVs in healthy donors, OU patients and OSCC patients were 45.1 ± 14.0%, 42.6 ± 8.2% and 49.2 ± 20.5%, and the percentages of non‐apoptotic SMVs (CFSE^+^Annexin V^−^) SMVs were 54.9 ± 14.0%, 57.4 ± 8.2% and 50.8 ± 20.5% respectively (Figure 3B). There was no significant difference in the ratio of Annexin V^+^ to Annexin V^−^ SMVs among healthy donors, OU patients and OSCC patients (data not shown). These results suggest that apoptotic and non‐apoptotic SMVs are almost in equal proportion, with no significant change in OSCC patients. By contrast, we found that the majority of CMVs in OSCC patients were negative for Annexin V staining (Figure 3C). To investigate the significance of apoptotic SMVs in OSCC, we then examined the association of the ratio of Annexin V^+^ to Annexin V^−^ OSCC‐SMVs with clinicopathologic features of OSCC patients. Of interest, flow cytometric results (Figure 3D) showed that the percentage of Annexin V^+^ SMVs was significantly decreased in OSCC patients with a higher pathological grade (III) when compared to those with lower pathological grades (I and II, *P* < 0.01), consistent with the observation by fluorescence microscope (Figure 3E). These results suggest that the proportion of apoptotic SMVs is decreased while the proportion of non‐apoptotic SMVs is increased in OSCC patients with a higher pathological grade. Correspondingly, as shown in Figure 3F, the ratio of Annexin V^+^ to Annexin V^−^ OSCC‐SMVs had a negative correlation with pathological grade (*P* < 0.01). These above data demonstrate that the ratio of apoptotic to non‐apoptotic OSCC‐SMVs is decreased in poorly differentiated OSCC, suggesting that the formation mechanism of SMVs in terms of apoptosis is altered during the malignant progression of OSCC.

### 3.6 Prognostic significance of SMVs in OSCC patients

**Figure 4:**
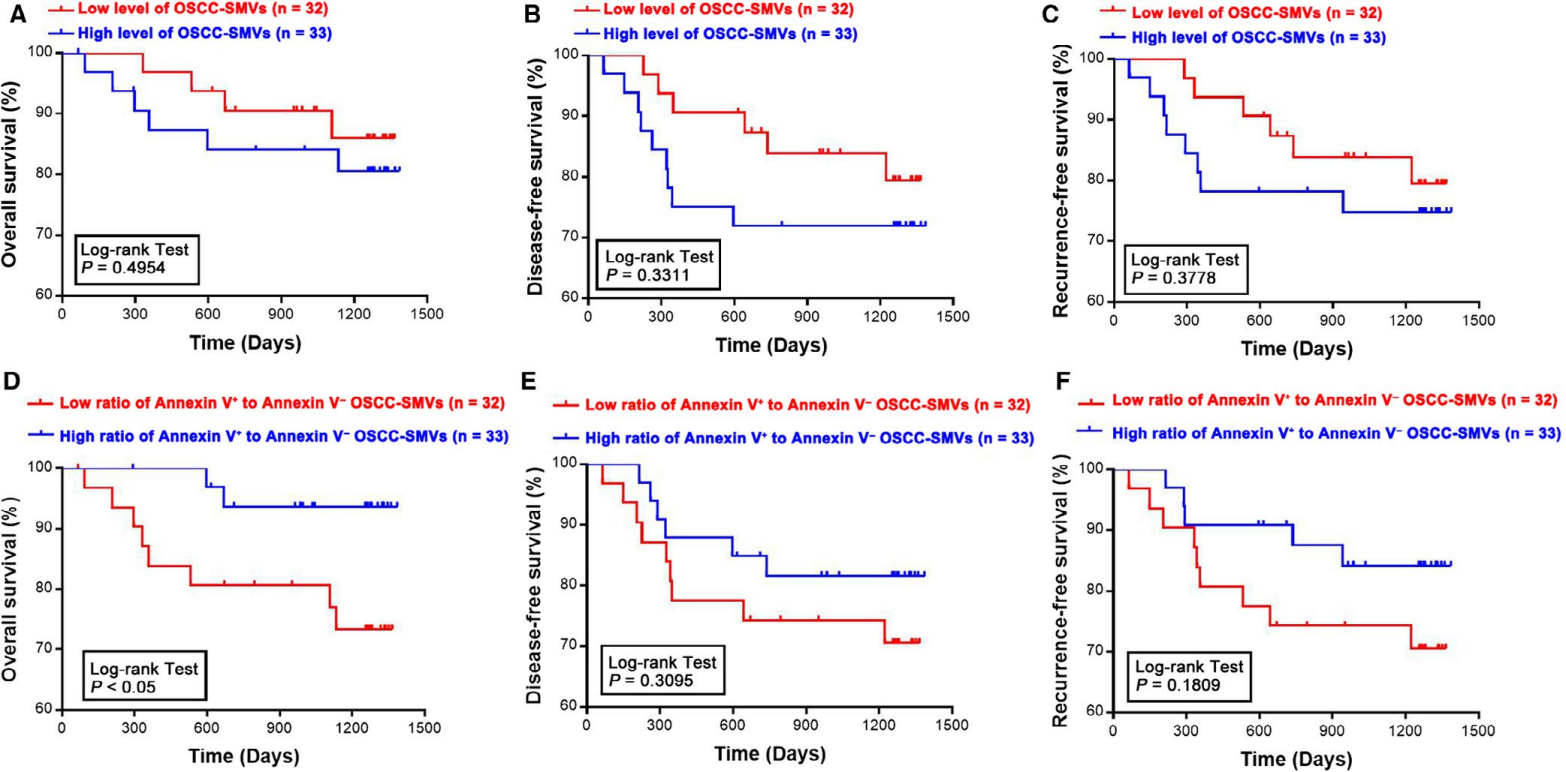
Prognostic values of salivary microvesicles (SMVs) in oral squamous cell carcinoma (OSCC) patients. Kaplan‐Meier analyses of the overall survival, disease‐free survival and recurrence‐free survival of OSCC patients were performed according to the level of total SMVs (A‐C) in patients with OSCC (OSCC‐SMVs) or the ratio of Annexin V^+^ to Annexin V^−^ OSCC‐SMVs (D‐F). n: the number of subjects in the group

To further determine the prognostic values of SMVs, patients with OSCC were divided into two groups based on either the level of OSCC‐SMVs or the ratio of Annexin V^+^ to Annexin V^−^ SMVs. Kaplan‐Meier analysis revealed that the overall survival, disease‐free survival and recurrence‐free survival curves stratified but showed no significant difference according to the level of total OSCC‐SMVs (Figure 4A‐C). Also, these survival curves stratified based on the ratio of Annexin V^+^ to Annexin V^−^ OSCC‐SMVs (Figure 4D‐F). With a statistical significance, OSCC patients with a higher ratio of Annexin V^+^ to Annexin V^−^ OSCC‐SMVs were predicted to have a better outcome in terms of overall survival (*P <* *0.0*5). These above results suggest the prognostic value and biomarker potential of SMVs, especially the ratio of Annexin V^+^ to Annexin V^−^ SMVs.

## 4 DISCUSSION

Previous studies have showed that the level of MVs, which are submicron (100‐1000 nm) membrane vesicles released by cells upon apoptosis or activation, is elevated in various human body fluids due to the development of malignant tumours.^11^, ^31^, ^32^ Although the presence of MVs in saliva of healthy donors has been reported by a previous study,^13^ the association between SMVs and OSCC is still unknown. In the present study, we find that the level of SMVs is significantly up‐regulated in patients with OSCC compared to healthy donors and OU patients. Meanwhile, the level of SMVs shows positive correlation with the clinicopathologic features of OSCC patients. Moreover, the ratio of Annexin V^+^ to Annexin V^−^ SMVs, which represents the ratio of apoptotic SMVs and non‐apoptotic SMVs, is significantly decreased in patients with poorly differentiated OSCC. More importantly, OSCC patients with higher ratio of apoptotic to non‐apoptotic SMVs show better overall survival than patients with lower ratio of apoptotic to non‐apoptotic SMVs.

Recently, our studies have shown that the level of CMVs is elevated and closely associated with the increased procoagulant activity and tumour angiogenesis in OSCC patients.^8^, ^9^ Here, we found that the major subpopulations and the formation mechanism (in terms of apoptosis) of SMVs and CMVs in OSCC patients were different. CMVs derived from OSCC patients were almost platelet‐derived MVs, while more than 50% of SMVs derived from OSCC patients showed epithelial origin. Moreover, consistent with a previous report,^33^ we found that the majority of CMVs derived from OSCC patients were negative for Annexin V staining (indicative of non‐apoptotic MVs), while apoptotic and non‐apoptotic SMVs were almost in equal proportion in OSCC patients. These findings suggest that the levels of SMVs and CMVs are probably elevated in OSCC patients through different mechanisms. Our previous study has revealed that the elevated level of CMVs in OSCC patients shows a close correlation with the secretion of inflammation‐related cytokines by OSCC cells. Although the mechanism behind the elevated level of SMVs in OSCC patients remains to be investigated, it is a reasonable hypothesis that MVs secreted by apoptotic and/or activated OSCC cells or other cells in the tumour microenvironment may contribute to the increase in the level of SMVs. As collection of saliva samples is convenient, noninvasive and low‐cost, SMVs may possess unique advantages over CMVs to serve as potential biomarkers for OSCC.

It has been widely accepted that both cell apoptosis and activation can result in the generation of MVs.^29^ To investigate the clinical significance of SMVs in terms of apoptosis in OSCC patients, we performed Annexin V staining by flow cytometry, which has been widely used to distinguish between apoptotic (Annexin V^+^) and non‐apoptotic (Annexin V^−^) MVs.^6^, ^14^, ^34^ We found that the ratio of apoptotic to non‐apoptotic SMVs possessed a negative correlation with the pathological grade in OSCC patients, and OSCC patients with lower ratio of apoptotic to non‐apoptotic SMVs were predicted to have a worse prognosis. These data suggest that SMVs may be a promising diagnostic and prognostic biomarker for OSCC patients.

So far, VEGF‐C is considered to be a major pro‐lymphangiogenic growth factor that can up‐regulate lymphatic vessel density and promote the tumour cells metastasize to lymphatic node.^35^, ^36^ Interestingly, our results revealed that the level of SMVs possessed a positive correlation with the expression level of VEGF‐C in OSCC tumour samples, suggesting a close association between SMVs and the lymph node metastasis of OSCC. Previous studies have documented that platelet‐derived MVs, the main CMVs, could transfer the receptor to cancer cell surface, induce kinase‐dependent protein phosphorylation and elevate the level of the matrix metalloproteinases to enhance cellular invasion.^37^, ^38^ In our study, although the precise mechanism behind the promotive effects of SMVs on OSCC cells in the VEGF‐C expression remains to be elucidated, our findings suggest that increased SMVs is not only a potential biomarker of clinicopathologic features of OSCC patients but may be also functionally involved in the development of OSCC, such as lymph node metastasis.

In summary, our present study reports that the level of SMVs is elevated in patients with OSCC, and the increased level of SMVs is closely correlated with the clinical data and outcomes of OSCC patients. Moreover, we also demonstrate that the ratio of apoptotic to non‐apoptotic SMVs is significantly decreased in patients with poorly differentiated OSCC. Importantly, lower ratio of apoptotic to non‐apoptotic SMVs predicts poorer overall survival of OSCC patients. Taken together, this study highlights the biomarker potential and functional significance of SMVs in OSCC.

## CONFLICTS OF INTEREST

The authors confirm that there are no conflicts of interest.

## AUTHOR CONTRIBUTIONS

WQ Zhong, YF Zhao and G Chen participated in the conception design, data acquisition and analysis, and wrote the manuscript. WQ Zhong, JG Ren and QW Man performed the experiments. XP Xiong, B Liu, ZJ Sun, J Jia and WF Zhang helped with the collection of clinical samples. W Zhang and G Chen revised the manuscript. All authors approve and agree to be responsible for all aspects of this work.
