# Supplementary figures and images for: Increased salivary microvesicles are associated with the prognosis of patients with oral squamous cell carcinoma
Source: J Cell Mol Med. 2019 Mar 25;23(6):4054–62. doi: 10.1111/jcmm.14291 (PMC6533497; doi:10.1111/jcmm.14291)

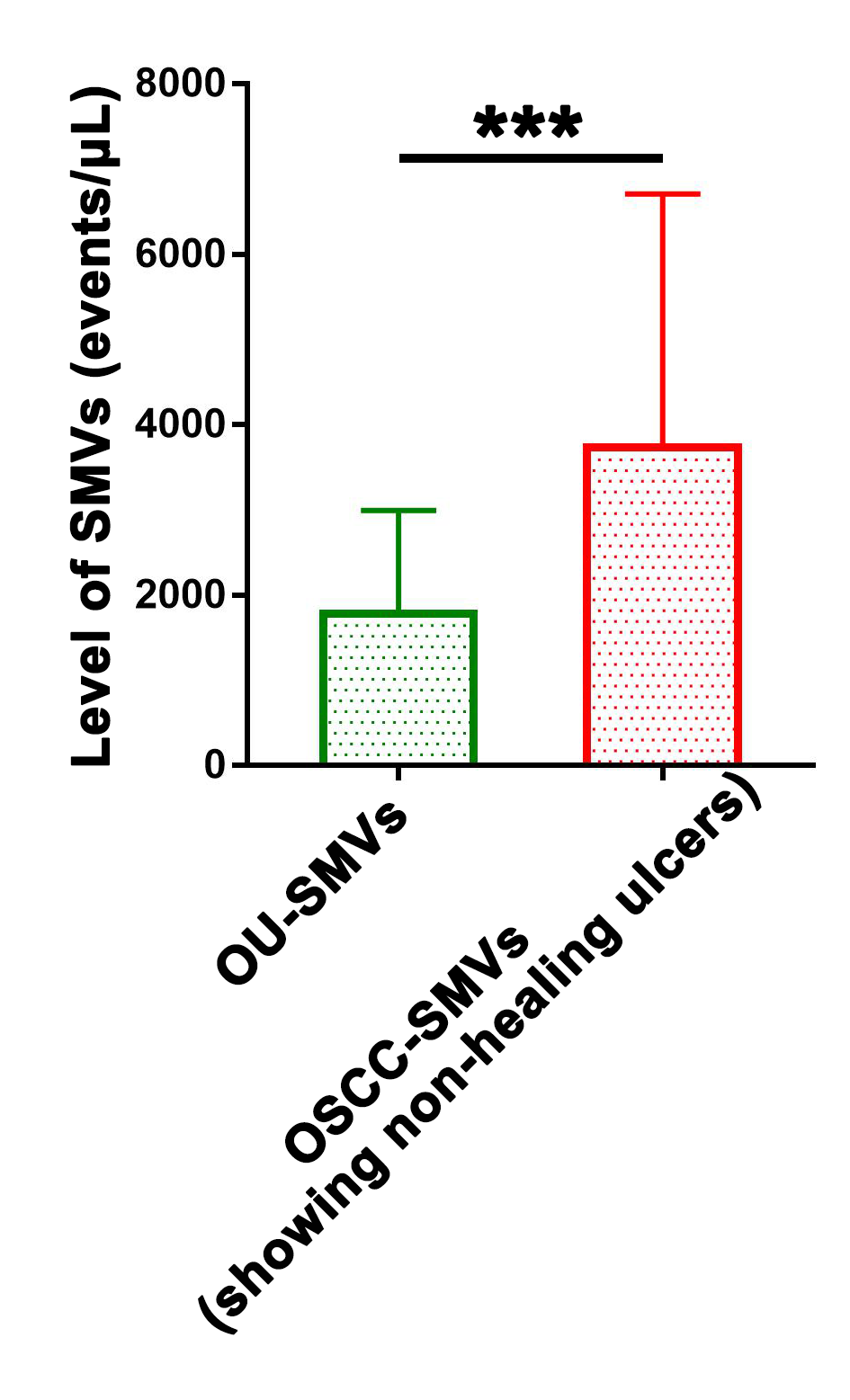

Supplement: Supplementary file 2 [file JCMM-23-4054-s002.tif]
